# Communication disorders in individuals with cleft lip and palate: An overview

**DOI:** 10.4103/0970-0358.57199

**Published:** 2009-10

**Authors:** Roopa Nagarajan, V. H. Savitha, B. Subramaniyan

**Affiliations:** Department of Speech Language & Hearing Sciences, Sri Ramachandra University, Chennai, India

**Keywords:** Articulation, Cleft lip and palate, Communication disorder, Resonance, Velopharyngeal dysfunction

## Abstract

The need for an interdisciplinary approach in the comprehensive management of individuals with cleft lip and palate is well recognized. This article provides an introduction to communication disorders in individuals with cleft lip and palate for members of cleft care teams. The speech pathologist is involved in identifying those infants who are at risk for communication disorders and also for initiating early intervention to prevent or mitigate communication disorders caused by the cleft. Even with early cleft repair, some children exhibit ‘cleft palate speech’ characterized by atypical consonant productions, abnormal nasal resonance, abnormal nasal airflow, altered laryngeal voice quality, and nasal or facial grimaces. These manifestations are evaluated to identify those that (a) are developmental, (b) can be corrected through speech therapy alone, and, (c) those that may require both surgery and speech therapy. Speech is evaluated perceptually using several types of stimuli. It is important to identify compensatory and obligatory errors in articulation. When velopharyngeal dysfunction is suspected, the assessment should include at least one direct measure such as nasoendoscopy or videofluoroscopy. This provides information about the adequacy of the velopharyngeal valve for speech production, and is useful for planning further management of velopharyngeal dysfunction. The basic principle of speech therapy in cleft lip and palate is to establish the correct placement of the articulators and appropriate air flow. Appropriate feedback is important during therapy for establishing the correct patterns of speech.

## INTRODUCTION

Individuals with cleft lip and palate often demonstrate multiple problems such as early feeding difficulties, nutritional issues, developmental delays, abnormal speech and / or resonance, dentofacial and orthodontic abnormalities, hearing loss, and possibly, psychosocial issues.[1] A coordinated team approach involving follow-up from infancy to adulthood is recommended for optimal outcomes.

In the past decade, there has been a significant increase in the number of surgeons who provide services for individuals with cleft lip and palate in India. It is well accepted that the primary purpose of palate repair is to facilitate speech production.[2,3] Thus, there has been great interest in the measurement of surgical outcomes, candidacy for secondary surgeries for speech, and speech therapy. Speech-language pathologists evaluate language, speech, resonance, and velopharyngeal function in individuals with cleft lip and palate and make recommendations for appropriate treatment. They also provide intervention for communication disorders. This article provides an orientation for members of cleft care teams on speech and language disorders in this population.

Individuals with cleft lip and palate form a diverse group varying from those who have an isolated cleft, to those in whom cleft lip and palate is more of a feature of a syndrome. This diversity makes it difficult to make generalizations about the characteristic features of communication disorders in those with cleft lip and palate. D'Antonio and Scherer listed several factors: type and severity of cleft, age and time of palate repair, efficacy of repair, unrepaired residual cleft, presence of fistula, status of velopharyngeal function, hearing status, and socioeconomic and linguistic status that could impact communication in this population.[4] They also emphasized the need to keep a developmental perspective in mind as the management (surgical, dental, or speech) is timed with reference to physical growth and development.

The first priority for a child born with cleft lip and palate is feeding. This is to ensure that the child meets the nutritional and growth requirements needed for surgery. There has been much debate and research in developed countries on delineating the most appropriate timing for palate repair that would be optimal for the development of speech without compromising orofacial growth.[5] In many regions of the developing world, late palate repair is the rule rather than an exception because of the paucity of trained manpower and resources. A child with a cleft of lip and palate may never be referred to a speech pathologist, or be referred only if and when a problem in communication becomes evident. Furthermore, speech pathology may be nonexistent as a profession or be in its infancy in many of these countries.

## EARLY LANGUAGE DEVELOPMENT IN CHILDREN WITH CLEFT LIP AND PALATE

There have been several reports that children with cleft lip and palate show delayed expressive language, evidenced by slower acquisition of sounds and words and restricted inventory of sounds in early infancy.[6,7] It was also believed that they catch up by the age of three, usually after palate repair. Recently, a number of studies have documented that the children with cleft lip and palate show delays in language development that may encompass both receptive (comprehension) and expressive (production) language.[8] Some reports suggest that these early difficulties in the acquisition of language may persist into childhood in some individuals.[9,10] In the light of such evidence, it is important for infants and young children with repaired / unrepaired cleft lip and palate to be assessed for language development.

In the past decade, there has been a paradigm shift in the approach towards the management of communication disorders in very young children with cleft lip and palate. There is an increasing emphasis on the identification of “at risk” children. The recognition of differences in language and speech development in infants and young children with cleft lip and palate has resulted in several investigators exploring early intervention models that incorporate language stimulation techniques involving play therapy and parent infant programs.[11] It has been demonstrated that these intervention models not only improve language skills, but also promote speech sound production. Scherer, D'Antonio, and McGahey reported reduction in compensatory errors,[12] a finding that has important implications in underserved regions of the world where a “prevention model” may be easier and more practical to implement.

## EFFECTS OF CLEFT ON SPEECH

Even with early surgical repair, a majority of preschoolers demonstrate delays in speech sound development and have typical cleft palate speech.[13] The term, ‘cleft palate speech’ is used to describe phenomena such as atypical consonant productions, abnormal nasal resonance, abnormal nasal airflow, altered laryngeal voice quality, and nasal or facial grimaces.[14] A profile of communication disorders in 129 individuals with repaired cleft of lip and / or palate above the age of three years from a district in South India, revealed that 38% had normal and age-appropriate communication skills. The majority of those with normal communication skills had isolated cleft of the lip. Forty-three percent of the 129 individuals exhibited abnormalities in articulation and resonance, 12% had only articulation deviations, and 3% only abnormalities in resonance. Another 3% of these individuals exhibited delays in language development.[15] In order to appreciate the effect of cleft on speech, it is essential to understand the mechanism of speech production.

### Normal speech production

The production of speech involves a series of coordinated movements that begins with airflow from the respiratory system. This airflow is modulated at the laryngeal, articulatory, and resonatory systems, producing different sounds. For precise production of various sounds, it is also necessary to have effective feedback and control, which requires an intact auditory and neuromotor system. Various sounds are produced across the vocal tract, depending on the place and the manner of these modulations. The vowel sounds are produced without any significant constriction made by the tongue / lip, and are classified based on the position and height of the tongue and rounding of the lips. For example, the vowel /a/ as in ‘arch’ is described as a low mid vowel; /i/ as in ‘inch’ is described as a high front vowel; while /u/ as in ‘soup’ is a rounded high back vowel. The consonants are classified as glottal, pharyngeal, velar, palatal, retroflex, alveolar / dental, labiodental, and bilabial, based on the place of articulation, *i.e*., the place where a constriction is made by the tongue / lip. Table 1 summarizes the classification of consonants based on the manner of articulation. Consonants are further classified as voiced or voiceless, depending on the modulation at the level of vocal folds. For example /p/ as in ‘pat’ is a voiceless bilabial stop and /b/ as in ‘bat’ is a voiced bilabial stop; /s/ as in ‘sit’ is a voiceless alveolar fricative; /m/ as in ‘mat’ is a voiced nasal continuant.

**Table 1:** Classification of speech sounds based on manner of articulation

*Manner of articulation*	*Modulation*	*Example*
Stops	Completely blocking the air behind the constriction made by the tongue/lip and releasing it suddenly.	/p/ as in pen, /b/ as in bag, /t/ as in tea, /k/ as in king
Fricatives	Narrow constriction with air rushing through the narrowed passage.	/s/ as in see, /z/ as in zebra
Affricates	Combination of stops and fricatives. Produced by complete closure made by the tongue followed by slow release of air through a narrow constriction.	/ch/ as in chips, /j/ as in jug
Laterals	Closure in the midline of the tongue with lateral escape of air.	/l/ as in look
Trills/Rhotics	Vibration of the tip of the tongue against the air stream.	/r/ as in read
Glides	Produced by gliding, or gradually changing the shape of the articulators	/w/ as in watch

### Errors in speech sound production

In individuals with cleft lip and palate, errors in speech production are noticed due to the abnormalities in oronasal structure / function, orofacial structure and growth, learned neuromotor patterns during early infancy, and / or disturbed psychosocial development.[16] A wide variety of speech sound errors are noticed in these individuals. Comparatively, the pressure consonants (stops, fricatives and affricates) are more affected than the other sounds. Henningsson *et al*. summarized the various atypical consonant productions that can be observed in individuals with cleft lip and palate.[17] Table 2 summarizes the common error patterns seen in individuals with cleft lip and palate.

**Table 2:** Common error patterns noticed in individuals with Cleft Lip and Palate

Abnormal backing of oral targets to postuvular place (to pharyngeal or glottal)
Abnormal backing of oral targets within the oral cavity (to uvular or velar or palatal)
Nasal fricatives
Nasal consonants for oral pressure consonants
Nasalized voiced pressure consonants
Weak oral pressure consonants
Other misarticulations involving lateralized or palatalized productions of fricatives
Developmental articulation/Phonological error

Broadly, cleft type errors of speech sound production are classified into two types: obligatory and compensatory.[18] Obligatory errors include errors in production due to interference of structural abnormalities, such as malaligned tooth, residual clefts, oronasal fistula, etc. They generally result in changes in the manner of articulation. These errors cannot be corrected through speech therapy unless the underlying structural deformity is corrected. Compensatory errors include errors that occur due to maladaptive articulatory placements learned by children during the developmental period. These errors involve changes in the placement of articulation, with the manner being retained. For example, a stop consonant is produced as a stop, but with a posterior placement of the articulator (/p/ produced as a /k/ or /t/). These errors can be corrected only through speech therapy. It has been demonstrated that the prognosis and outcomes of therapy are better if the intervention is started early.[19]

It is important to assess speech sound production across linguistic levels (words, sentences, and conversation).[17] It is not uncommon for a sound to be produced accurately at the word level and be in error only at the sentence level. Appropriate care should be taken to develop the controlled single-word and sentence stimuli (with emphasis on pressure consonants) that can be elicited in individuals with cleft lip and palate. In India, controlled speech stimuli have been developed in Tamil, Malayalam, and Kannada. However, there is a need to develop such materials across other Indian languages as well. This would enable uniform protocols for the assessment of speech production in individuals with cleft lip and palate.

### Abnormal nasal resonance and airflow

Abnormal nasal resonance is another characteristic feature in most individuals with cleft lip and palate. The resonance of speech is largely determined by the size and shape of the oral, nasal, and pharyngeal cavities, and the functioning of the velopharyngeal valve. The abnormal nasal resonance in cleft lip and palate could involve either hypernasality or hyponasality. Hypernasality refers to an excessive nasal resonance that is perceived for vowels and oral consonants. Hyponasality indicates a decreased nasal resonance for nasal consonants and vowels. In some individuals with cleft, hypernasality and hyponasality co-exist, resulting in a mixed nasality. Abnormal resonance can be caused by structural disturbances such as obstructions in the nasopharynx due to adenoid hypertrophy, swelling of the nasal passages secondary to allergic rhinitis, or hypertrophic tonsils (causing hyponasality), large oronasal fistula, and velopharyngeal dysfunction (causing hypernasality). Occasionally, hypernasality could be caused due to velopharyngeal mislearning, wherein only certain sounds are perceived to be hypernasal. This is referred to as phoneme-specific hypernasality, which occurs due to the incorrect placement of oral structures for certain sounds (*e.g*., ng/l, ng/r).[20]

Conditions such as large oronasal fistula and velopharyngeal dysfunction (VPD) will also result in disturbed nasal airflow in addition to hypernasal resonance. These conditions result in air emission while attempting to produce pressure-sensitive sounds. The loudness of this emission is determined by the size of the opening (fistula or velopharyngeal port). Phoneme-specific nasal air emission can also be noticed due to velopharyngeal mislearning.[20]

Very often, resonance and airflow disturbances in individuals with cleft lip and palate are due to VPD. Shprintzen reported that 10–20% of children undergoing primary palatoplasties before the age of 18 months have associated VPD.[21] The occurrence of VPD may be much higher in children who undergo primary palate repair at later ages.

## VELOPHARYNGEAL VALVING

The velopharyngeal valve is a tridimensional muscular valve that is located between the oral and nasal cavities. It consists of the lateral and posterior pharyngeal walls as well as the soft palate. The role of the velopharyngeal valve is to separate the oral and nasal cavities during speech and swallowing. During speech production, the air is directed through the mouth for oral sounds and through the nose for nasal sounds. VPD is the inability to separate the oral and nasal cavities adequately during speech production through the actions of the velum and pharynx.

### Velopharyngeal dysfunction (VPD)

The term, “VPD” is used in this article for consistency although it is recognized that other terminologies such as “palato-pharyngeal insufficiency”, “velopharyngeal inadequacy,” “velopharyngeal insufficiency,” etc. are also used.[22–24] The etiology of VPD could be structural, neurological, or learned. Velopharyngeal dysfunction can be caused due to lack of tissue (velopharyngeal insufficiency) or lack of proper movement (velopharyngeal incompetence) of the walls. While VPD is commonly associated with cleft lip and palate, it can also be seen with submucous cleft and other noncleft conditions such as ablative palatal lesions, adenoidectomy, deafness or hearing loss, and cerebral palsy.[20,25,24]

### Effects of VPD on speech

In VPD, the incompletely closed velopharyngeal valve causes an inability to effectively manage the air stream for continuous speech. This could be manifested as one or more of the following:

- Hypernasality
- Audible / visible nasal air emission during production of oral consonants like /p/b/t/d/
- Facial / Nasal grimaces
- Weak or omitted consonants
- Reduced mean length of utterance
- Compensatory articulation errors

### Assessment of VPD

The first step in the assessment of VPD involves a detailed perceptual evaluation. It is important to use speech stimuli across different levels including syllables, words, and sentences during the assessment. This would place a higher demand on the velopharyngeal functioning. The profile obtained through perceptual evaluation will provide some insight about the status of the velopharyngeal function and indicate whether instrumental evaluation such as nasoendoscopy and videofluoroscopy are needed.

Nasoendoscopy and videofluoroscopy are direct measures that permit the examiner to view the anatomical and physiological defects that cause VPD.[1,21] A standard set of speech tasks including spontaneous speech, repetition of oral and nasal consonants at syllable level (/papi/, /kaki/, /sasi/, /mami/), number-counting task in English (sixty to seventy and ninety to ninety-nine), and sentence repetition (sentences loaded with oral and nasal consonants) are used during both the procedures.

Nasoendoscopy is a minimally invasive procedure that requires compliance from the individual. It provides a view of all the structures of the velopharyngeal mechanism and determines the location, size, and shape of the velopharyngeal opening.[26] Compared to videofluoroscopy, nasoendoscopy helps more in the visualization of even small openings in the velopharyngeal valve. However, videofluoroscopy gives a better picture of the length of the velum and its upward movement during speech.[27,28] It also provides a view of the entire length of the posterior pharyngeal wall. The information from multiple views (frontal, basal, and lateral) has to be extrapolated to appreciate the functioning of the valve in all the three dimensions. Also, exposures to radiations in videofluoroscopy necessitate the procedure to be brief. Comparatively, videofluoroscopy is a less invasive procedure than nasoendoscopy.

Both nasoendoscopy and videofluoroscopy are well recognized procedures in clinical practice. It is important to perform at least one of them when warranted, so that information on the pattern of velopharyngeal closure for speech can be obtained to decide on further treatment options. Information about the type of closure and nature of defect serves as a base in the differential diagnosis and decision-making for treatment.

Nasometry is another procedure used for the assessment of resonance. It is an indirect measure of resonance as it does not allow the clinician to visualize the velopharyngeal port. It is a noninvasive procedure that measures the oral and nasal energy during a speech task. The output of this instrument is a numerical score that reflects the nasal acoustic energy in an individual's speech. These values are then compared to the standardized norms for that particular language for interpretation. Normative data on nasalance scores are available in some Indian languages.[29] It is well accepted that the information obtained can be used for pre- and posttreatment comparisons but not to make decisions for treatment options.

### Management of VPD

The treatment of velopharyngeal dysfunction may include surgical intervention (pharyngeal flap, sphincter pharyngoplasty, or posterior pharyngeal wall augmentation) and speech therapy. Prosthetic devices can also be used on a temporary or permanent basis in some instances. Surgery is often needed to correct the structural deficit, but does not necessarily result in change of function. Therefore, postoperative speech therapy is usually indicated to help the individual make the best use of the surgical correction, and also, to correct the compensatory articulation errors associated with VPD.

## EFFECTS OF CLEFT ON VOICE

Individuals with cleft lip and palate may also exhibit dysphonia. This is characterized by breathiness, hoarseness, and low intensity of voice during speech tasks. This is usually due to increased respiratory and muscular effort, and hyper-adduction of vocal folds while attempting to close the velopharyngeal valve.[20] The presence of dysphonia often masks nasality, making perceptual evaluation difficult.

## PRINCIPLES OF SPEECH THERAPY FOR INDIVIDUALS WITH CLEFT LIP AND PALATE

Speech intervention for individuals with cleft lip and palate can begin even before the palate is repaired. In very young infants, the emphasis is on training the parent / caregiver to stimulate the child's ability to understand and use language. Studies have demonstrated the efficacy of early intervention models using parents in the prevention of compensatory errors.[12] There is a need to apply and analyze the efficacy of such programs in the Indian scenario.

Older children (about age three) can be involved in direct therapy for the correction of errors in speech sound production (misarticulation). The goals are to establish the correct placement of the oral structures for speech sound production and directing the airflow appropriately. Goals are set depending on the error patterns and the age of the child. It should be kept in mind that errors due to structural defects cannot be corrected through speech therapy unless the structural deformity is corrected. Also, the structural correction should invariably be followed with speech therapy to correct the functioning / production of speech sounds. Appropriate feedback (using multiple modalities such as auditory, visual, tactile, etc.) is extremely important in the management of articulation and resonance disorders.

Elements of oromotor therapy (such as blowing, sucking, whistling, and electrical stimulation) are not useful in facilitating the correct production of speech sounds.[30] It has been demonstrated that there are significant differences in the velopharyngeal closure patterns of speech and nonspeech activities. Furthermore, because individuals with cleft lip and palate have a structural deformity and not a muscle weakness, oromotor exercises should be avoided in this population.

## CONCLUSION

As a member of the cleft care team, the speech pathologist works closely with the surgeon and other team members to ensure that timely assessments and appropriate management are provided. The first assessment of communication skills should begin in infancy even before the child begins to speak, focusing on the language skills and emerging sound production. In older children, accurate assessment is required to identify those children who would benefit from speech therapy and / or secondary surgery for optimizing speech outcomes. Speech language therapy focuses not only on direct one-on-one therapy, but also on early intervention programmes that will reduce the manifestation of communication disorders in individuals with cleft lip and palate. Cleft care is most successful when services are not only comprehensive, but also interdisciplinary in nature. Thus, it is important for each member of the team to understand the fundamental principles of care in the area of expertise of other members of the team.
